# Distributed abstraction and verification of an installed optical fibre network

**DOI:** 10.1038/s41598-021-89976-w

**Published:** 2021-05-24

**Authors:** D. J. Ives, S. Yan, L. Galdino, R. Wang, D. J. Elson, Y. Wakayama, F. J. Vaquero-Caballero, G. Saavedra, D. Lavery, R. Nejabati, P. Bayvel, D. Simeonidou, S. J. Savory

**Affiliations:** 1 University of Cambridge, Cambridge, UK; 2 University of Bristol, Bristol, UK; 3 University College London, London, UK; 4 KDDI Research, Inc., Fujimino, Japan; 5 Universidad de Concepción, Concepción, Chile

**Keywords:** Optics and photonics, Electrical and electronic engineering

## Abstract

The management of wavelength routed optical mesh networks is complex with many potential light path routes and numerous physical layer impairments to transmission performance. This complexity can be reduced by applying the ideas of abstraction from computer science where different equipment is described in the same basic terms. The noise-to-signal ratio can be used as a metric to describe the quality of transmission performance of a signal propagated through a network element and accumulates additively through a sequence of such elements allowing the estimation of end-to-end performance. This study aims to explore the robustness of the noise-to-signal ratio metric in an installed fibre infrastructure. We show that the abstracted noise-to-signal ratio is independent of the observers and their location. We confirm that the abstracted noise-to-signal ratio can reasonably predict the performance of light-paths subsequently set in our network. Having a robust network element abstraction that can be incorporated into routeing engines allows the network management controller to make decisions on the most effective way to use the network resources in terms of the routeing and data coding format.

## Introduction

Optical fibre networks form the backbone of the entire digital communications infrastructure and the Internet^1^. The data is encoded onto optical carriers that are guided within the optical fibres to propagate from source to destination. The optical fibres are linked by re-configurable optical add drop multiplexers (ROADMs)^2^ forming an optical mesh network connecting metropolitan areas, continents and the globe. Standard single-mode optical fibre can simultaneously guide multiple optical signals distinguished by their wavelength. The ROADMs are set to switch different wavelengths between different fibres allowing the different signals to follow different routes and arrive at their intended destinations. This gives us a transparent wavelength routed optical network (WRON)^1^ where the optical path from source node to destination node is determined by the transmitted optical wavelength and the ROADM switch configurations.

The management of such a WRON is complex, the determination of a large number of non-conflicting routes and wavelengths to satisfy the data transport demands is computationally hard^3^. We must add to this basic complexity two additional features for future WRONs. Firstly the use of flexible networking where the signal bandwidth and bit rate can be flexibly adjusted to match the client data requirements^4,5^. Secondly the analogue nature of the optical signals in a transparent WRON leads to an accumulation of noise and interference as the signal propagates. These impairments lead to a decrease in the quality of transmission^6^ restricting the maximum data rate with guaranteed error free transmission.

Traditionally the performance of a light-path was estimated with vendor proprietary tools and a system margin of 2 dB^7^ added to mitigate unknowns and errors. The Gaussian noise (GN) model has been shown to accurately predict experimental performance to 0.75 dB^8^ while adapting the GN model parameters through measured performance has reduced this to 0.6 dB^9^. Alternative approaches using machine learning^10^ both directly and as a way to optimise physical layer models show excellent results with emulations < 0.1 dB^11^ but require considerable amounts of training data.

Applying the ideas from computer science the complexity of the optical network can be simplified by combining individual optical components into network elements that are abstracted and described by a limited number of basic parameters. Software defined networking (SDN) has brought abstraction to the network switching elements^12^. To allow for impairment aware network control we must also abstract the network links, for example the optical links can be described in terms of start and end connectivity, length (latency), available bandwidth and a quality of transmission parameter. Such an abstraction provides a simplified view of the complex optical transmission to the network management control.

We choose to use the added noise-to-signal ratio (NSR)^13,14^ as our transmission metric assigned to each network element. This is the linear ratio between the worst case additive noise, including nonlinear interference (NLI) when the network element is handling the design channel load, from transmission through the element and the optical signal power. We calculate the noise within the receiver matched filter bandwidth since provided the signal power spectral density is fixed the NSR will remain constant to first order for changes in symbol rate. Under the assumption that the performance impact of the additive noise from different network elements is constant and uncorrelated the overall NSR for a path through the network can be estimated as the sum of NSR for all the elements through which the signal passes. This abstracted performance metric can be incorporated into the routeing tools to jointly optimise routeing, data coding and transmission performance. The NSR metric can be initially calculated from the optical component specifications or estimated at commissioning from probing transmissions^15,16^. Subsequently the metric can be maintained from network monitoring^17^. In this paper we use NSR to refer to the property of a network element and signal-to-noise ratio (SNR) to refer to the property of a signal.

Estimates of amplified spontaneous emission (ASE) noise were used as an optical signal-to-noise ratio (OSNR) metric to inform routeing through inverse addition in a small network^13^. The actual link parameters of a single channel point to point link have been estimated by probing^15^. This was extended^16^ to an installed fibre plant with heterogeneous span lengths where a single channel probe was used to abstract the links and used to predict performance of a multi-channel dense wavelength division multiplexed (DWDM) transmission. In the work^18^ three partners at three different locations abstracted an installed wavelength routed optical network using heterogeneous transmission equipment, equivalent to a multi-vendor network. The key novelties of this paper are to more fully demonstrating the capability over an installed wavelength routed optical network with inter-laboratory transmission and the reduction of inter-laboratory variation through calibrating of the performance monitoring capability of the disparate receivers against known ASE noise loading. This demonstrates that a simple abstraction can achieve comparable results to more complex techniques in a realistic scenario making it useful to transform resource allocation and network management.

## Results

**Figure 1:**
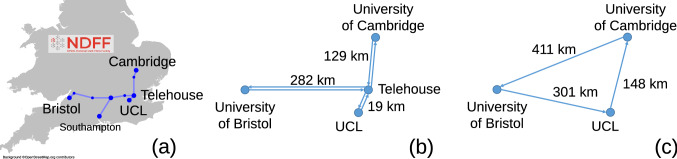
Network topology. The installed NDFF (**a**), the configured physical topology (**b**), and the virtual topology (**c**).

The experiments were carried out between the three partners over the UK national dark fibre facility (NDFF)^19^. This is an installed network between the University of Cambridge (UoC), University of Bristol (UoB) and University College London (UCL) as illustrated in Fig. 1a. The network was physically configured as a star network of three bidirectional links radiating from the space switch at Telehouse (Thn) as illustrated in Fig. 1b. The network was designed to operate with just 16 channels of approximately 32 GBd spaced on a 50 GHz grid.

**Table 1:** Loop-back NSR.

Looped at	From UoC	From UoB	From UCL
Thn	$2\ NSR_{C-T}$	$2\ NSR_{B-T}$	$2\ NSR_{U-T}$
UoC		$2\ NSR_{B-T} + 2\ NSR_{C-T} + NSR_{UoC}$	$2\ NSR_{U-T} + 2\ NSR_{C-T} + NSR_{UoC}$
UoB	$2\ NSR_{C-T} + 2\ NSR_{B-T} + NSR_{UoB}$		$2\ NSR_{U-T} + 2\ NSR_{B-T}+ NSR_{UoB}$
UCL	$2\ NSR_{C-T} + 2\ NSR_{U-T} + NSR_{UCL}$	$2\ NSR_{B-T} + 2\ NSR_{U-T} + NSR_{UCL}$	

Analytical NSR of each loop-back path after the NSR of the transceiver back to back through its local node has been removed.

The performance of the links within the physical network were probed by all three partners. Each partner transmitted 8 channels of around 32 GBd on a 50 GHz grid and monitored the quality of transmission. The quality of transmission was recorded for each partner for signals transmitted back to back through the local node only, looped back at the central Telehouse node and looped back at the two other partner laboratories. To give a consistent measure of the optical NSR within the network a noise loading calibration of each transceiver was carried out. The NSR measured on an optical spectrum analyser for an equivalent noise bandwidth equal to the symbol rate was compared to the transceiver measured performance.

We denote the NSR of the links for UoC to Thn, UoB to Thn and UCL to Thn as $NSR_{C-T}$, $NSR_{B-T}$ and $NSR_{U-T}$ respectively. Since the loop-back measurements are unable to distinguish between the NSR contributed by the two directions we assumed the NSR of the each direction in a link was equal. To separate the NSR of each element completely requires a full rank matrix of probing measurements. With limited measurements at the commissioning stage it will be necessary to make assumptions about symmetry or physical properties until sufficient light paths have been measured to obtain a full rank matrix. The NSR of the nodes for through routed signals is denoted $NSR_{UoC}$, $NSR_{UoB}$ and $NSR_{UCL}$ for the nodes at UoC, UoB and UCL respectively. The NSR of nodes associated with signals added is included in the transmitter NSR, while signals that are dropped are merely attenuated so gain no further noise. For signals that are not shot-noise limited attenuation reduces both signal and noise equally and does not change the SNR. Table 1 shows the expected NSR of measurements of each loop-back experiment for each participant, after removal of the transceiver back-to-back NSR, as a combination of the NSR for each network element as denoted above.

We solve globally to obtain estimates of the NSR of each node for through routed signals, constraining NSR to be $\ge 0$. This gave $NSR_{UoC}=0.0000$, $NSR_{UoB}=0.0014$ and $NSR_{UCL}=0.0019$. Finally each partner estimate of the NSR of each link was obtained given the equations of Table 1.

The estimated NSR of the network elements was calculated from the 8 channel probe measurements and an increase of 5.5% was applied to all the link NSRs to take account of the additional NLI caused when the network was operated at the 16 channel design load. This NLI noise increase is a small correction and was estimated from the GN model^20^. Table 2 shows the estimated NSR of each link under the full 16 channel design load as measured by each partner. The NSR of the links agree within 0.9 dB between partners with a standard deviation of 0.3 dB. This larger variation in the abstracted NSR of the shortest link is a result of taking differences of noisy measurements for low NSR. Since NSR is accumulated in the linear domain when the NSR of other network elements are included the variation of accumulated NSR will be reduced in the log domain.

**Table 2:** Estimated NSR (dB) of network links, at 16 channel full load^21^.

Measure at	Link UoC-Thn	Link UoB-Thn	Link UCL-Thn
UoC	− 24.4	− 23.5	− 26.2
UoB	− 24.6	− 23.2	− 27.1
UCL	− 24.8	− 23.3	− 26.4

For the verification of our abstraction we configured the physical network as a virtual single direction ring topology, as illustrated in Fig. 1c. Each partner could add/drop signals transported over 1, 2 or 3 virtual links. The links were all loaded with the designed 16 channels.

Using the estimated NSR of the physical links from Table 2 we calculate the NSR of the virtual links as estimated by each partner and shown in Table 3. We see that the errors associated with the NSR of the physical links are not independent since the NSR of the virtual links are in better agreement and are within 0.5 dB between partners with a standard deviation of 0.1 dB.

**Table 3:** Predicted NSR (dB) of virtual links, at 16 channel full load^21^.

Measure at	Link UoC-UoB	Link UoB-UCL	Link UCL-UoC
UoC	− 20.9	− 21.7	− 22.2
UoB	− 20.9	− 21.7	− 22.7
UCL	− 20.9	− 21.5	− 22.5
**Avg**	− 20.9	− 21.6	− 22.5
**Std**	0.1	0.1	0.2

To populate the virtual ring network, each partner launched three signals which propagate through one link, three signals that propagate through two links and two signals that propagate through all three links and return to their original node. UoC propagated one additional three link signal to populate all the links with 16 channels. The routeing and channel assignment used is illustrated in Table 6. Each signal was $\approx$32 GBd with PM-16QAM modulation allowing a net data rate of 200 Gbps per channel so that each link transports 3.2 Tbps.

**Figure 2:**
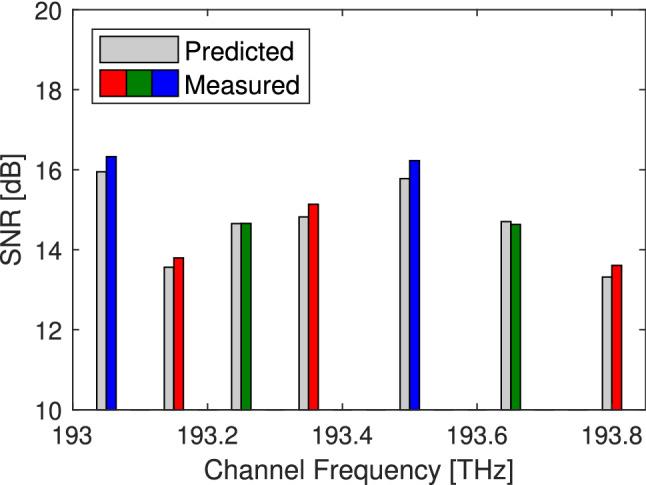
Received performance. Measured performance and predicted performance of signals propagated around the virtual ring. The performance histograms are grouped around the channel frequency. Signals transmitted from UoC, UoB and UCL are coloured red, green and blue respectively^21^.

As an initial verification we consider the three link signals that propagate fully around the network returning to their initial node. Figure 2 shows the measured and predicted performance for the seven signals propagating through three links. The difference between the measured and predicted performance ranges between 0.45 and − 0.07 dB. The average measured performance is better than the predicted performance, as expected since the abstraction is a worst case, however the experimental uncertainties lead to a small negative margin in one case such that a small system margin would be required to guarantee performance.

We now consider the performance of the signals propagated only part way around the loop between different partner laboratories. Firstly the signals from UoC measured at UCL where found to be almost 1 dB worse than expected and this was believed to be an error in one of the digital signal processing (DSP) parameter, as such the traces from UCL were re-analysed using DSP at UoC. This gave similar performance for the signals transmitted from UCL but more realistic performance for signals from UoC and UoB. Secondly it appears that signals which propagate over the link Thn-UCL have a better than expected SNR performance while those that propagate over the link UCL-Thn have poorer than expected performance. This suggests that while the abstraction correctly identified the total NSR for bidirectional propagation there is some significant asymmetry within this link, believed to be due to an exceptional high loss within UCL. Thirdly when transmitting between different partners it is necessary to separate the NSR due to the transmitter and that due to the receiver. Finally it was noted that isolated channels from UCL show worse performance than the grouped channels. This was identified as a result of a frequency offset within the wavelength selective switch (WSS) at UCL resulting in a switch edge allowing dropped channels to interfere with the transmitted signal. In the case of the grouped channels there is no switch edge within the bandwidth of the central and higher frequency channel, giving these channels better SNR performance. No such cross talk was observed from the WSS at UoC or UoB where the frequency grid of the WSS aligned more closely. Within this experiment, where the frequency was aligned, the guard bands and limited number of ROADMS ensured that WSS filtering effects did not have a significant impact on the signal quality. Consequently the effects of WSS frequency offset, and more general WSS filtering effects, have not been considered in this work and are left for future investigation.

Considering all these issues the NSR of the link from UCL to Thn was increased by 0.002 while that from Thn to UCL was reduced by 0.002. This leads to the NSR of the link from Thn to UCL to be more closely aligned with the design expectation putting all the additional noise associated with the excess loss onto the UCL to Thn link. UoC and UCL each use a single research coherent receiver while UoB had four commercial receivers, the NSR of these three receiver types was adjusted by hand to balance the results across all the measured signals, while the transmitter NSR was calculated from the back to back measurements. Table 4 shows the adjusted receiver NSR along with the typical transmitter NSR.

**Table 4:** Adjusted receiver NSR (dB) for each partner along with a typical transmitter NSR (dB).

Partner	Rx NSR (dB)	Tx NSR (dB)
UoC	− 23.5	− 18.5
UoB	− 22.5	− 22.2
UCL	− 33.5	− 24.5

**Figure 3:**
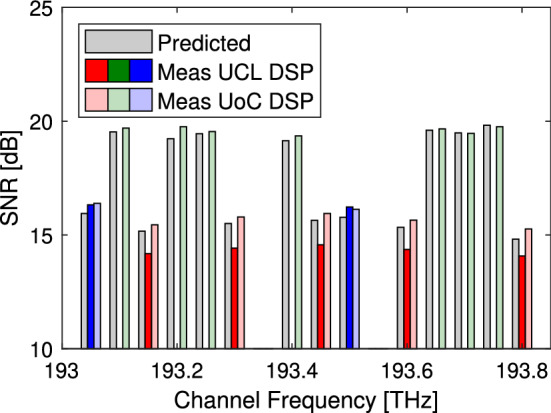
Received performance. Measured performance and predicted performance of signals received at UCL. The performance histograms are grouped around the channel frequency. The bold colours correspond to signals analysed with UCL DSP while the pale colours are the same data captures processed with UoC DSP. Signals transmitted from UoC, UoB and UCL are coloured red, green and blue respectively^21^.

**Figure 4:**
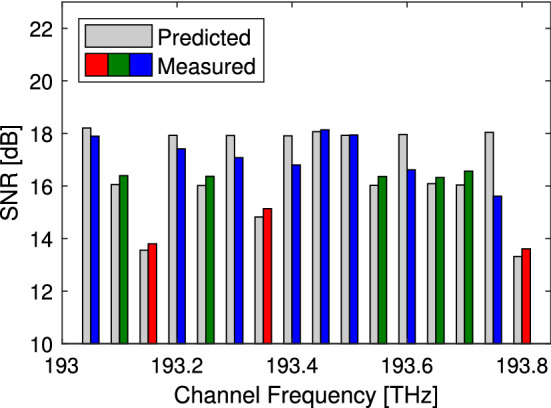
Received performance. Measured performance and predicted performance of signals received at UoC with UoC DSP. The performance histograms are grouped around the channel frequency. Signals transmitted from UoC, UoB and UCL are coloured red, green and blue respectively^21^.

We now verify the performance of the signals arriving at UCL and UoC, having been transmitted through 1, 2, or 3 links. Figures 3 and 4 shows the measured and predicted SNR at the receiver. The predicted SNR was calculated as the inverse of the accumulated NSR from all the network elements along the lightpath, including the transmitter and receiver. Overall all the signals transmitted 1, 2 or 3 links, with the exception of UCL signals received at UoB which could not be recovered and UCL signals with cross talk from the WSS, were found to have a performance that was within + 0.52, − 0.07 dB of the abstracted expectation similar to the results shown in Fig. 2. The slight negative margin is likely a result of measurement uncertainty and a small operating margin will be required to ensure all signals are received error free.

## Discussion

We have discussed the idea of abstraction applied to optical networks as providing each network element with a unified set of metrics that can be used to describe the properties and performance of that element. We introduce the use of noise to signal ratio as a performance metric that can be simply accumulated for signal transmitted through multiple elements.

We have assumed the NSR of each element to have a constant additive impact on the signal transmission performance. This requires the signal power within each element and the Erbium doped fibre amplifier (EDFA) ASE noise to be constant regardless of routeing order. In our network experiment the signal power was locally controlled for each span ensuring the signal power was independent of routeing history and the EDFA were gain controlled to ensure constant ASE noise. To have a constant impact the effect of WSS filtering should be insignificant. We also assume NLI is independent of accumulated dispersion and modulation format and is uncorrelated between network elements. This assumption is equivalent to that when using the popular incoherent GN model with the additional assumption that the different receivers treat NLI similarly without employing compensation. The incoherent GN model tends to provide a worst case NLI for moderate lengths^20^. The reported results have made these assumptions both during probing and operation such that it is the change in error between these regimes that is important.

We have previously shown^15,16^ that by probing a network with a transmission signal we can estimate the NSR for each network element. In this work we have shown that multiple observers can independently abstract a network gaining agreement of the performance of each element within 0.9 dB. We have then demonstrated through a simple ring topology that the performance of a light path can be estimated from the abstracted NSR of each element with agreement to within + 0.52, − 0.07 dB. This demonstrates that the simple additive NSR approach can improve over traditional margin based approaches^7^ and give comparable accuracy to more complex methods^8–11^.

While this was a small network it attempted to capture key network properties and the results are an important step to demonstrate the capability of NSR abstraction over an installed wavelength routed optical network with heterogeneous span lengths and heterogeneous transmission equipment. We have shown that the NSR metric can be assigned to each network element and used to predict performance of subsequent light paths in new topologies. The use of three independent observers strongly suggests the NSR metric is independent of the equipment used to measure and abstract it and also independent of the location of the observer. Location independence allows the network elements to equivalently abstracted locally or centrally.

During these measurements a number of potential issues were encountered. Care is required to make observations that will allow the separation of NSR associated with Tx and Rx and also the separation of NSR from transmission in either direction through a link. Further study is required to fully understand the degrading effects of WSS filtering in particular where the filter centre is miss-aligned from the signal carrier frequency. We have reported the results of a one time experiment equivalent to network commissioning. It is anticipated that once the abstracted NSR is established the time evolution, including variations in the transceiver calibration, will be tracked by monitoring the performance of active light-paths.

## Methods

### Experimental details

**Figure 5:**
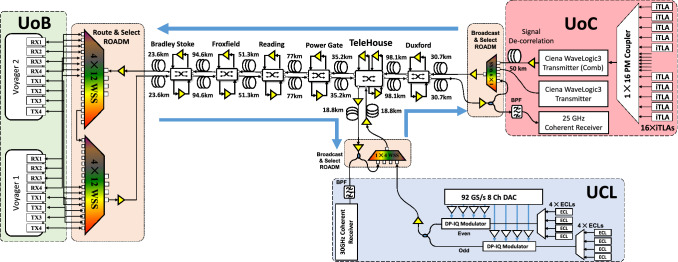
Experimental setup.

The experiment was performed over the NDFF^19^ and shown in Fig. 1a. Each intermediate node includes a number of EDFAs and a Polatis space switch to allow remote reconfiguration of the connections between fibres and amplifiers. To form a wavelength routed optical network each of the three universities constructed a degree one ROADM to attach to their fibre tails. A detailed illustration of the experimental setup is shown in Fig. 5.

Following previous experience^16,18^, the EDFA noise figure was assumed to be 5.75 dB and the fibre parameters assumed as shown in Table 5. The optimum launch power for each span was estimated from a worst case incoherent GN model assuming that the nonlinear interference for a single span was ${1}\big /{16}$ of that for 16 coherent spans of the same length. The launch power was further constrained by the capabilities of the EDFA. To maintain gain flatness across wavelengths the EDFA must operate at their design gain, as such in some spans it was necessary to reduce the launch power to avoid saturation of its following EDFA. The launch power into each span was set using the variable optical attenuator (VOA) within the Polatis switch at each intermediate node.

**Table 5:** System design and component parameters.

Parameter	Value	Unit
System		
Symbol rate	$\approx$ 32	GBd
WDM Grid Spacing	50	GHz
N^ọ^ Channels	16	
EDFA		
Noise Figure	5.75	dB
Fibre		
Attenuation Coeff	0.22	dB km$^{-1}$
Dispersion Coeff	16.4	ps nm$^{-1}$ km$^{-1}$
Nonlinear Coeff*	1.16	W$^{-1}$ km$^{-1}$

These values were assumed to apply across all 16 channels, 1550 ± 4 nm. *Effective nonlinear coefficient for launch power at switch monitor before any patch panel connector loss.

The University of Cambridge connected a broadcast and select ROADM to their fibre tails formed of a 50/50 coupler and a 1x9 WSS. Transmission signals were generated by two WaveLogic 3 modem cards with user programmed waveforms. One WaveLogic 3 had been modified to allow a user source to be injected before the modulator. A 16 laser array was injected into this modem card to generate 16 channels. These 16 channels were generated with a pre-dispersed waveform such that after passing through the transmitter integrated 50 km of standard single-mode fibre they emerge un-dispersed and time decorrelated. Signals were received by a 25 GHz integrated coherent receiver (ICR) connected to a 100 GS s^-1^ four channel real time oscilloscope. The data was recovered and performance monitored using offline DSP implemented in MATLAB. The SNR of the recovered constellations was estimated by comparing the measure constellation with the probability distribution of an ideal constellation for a given SNR and setting the SNR to maximise the likelihood of obtaining the measured constellation. A 10% pick off was fitted after the launch amplifier to allow monitoring of the transmitted waveform and the overall transmitted spectrum.

The University of Bristol connected a route and select ROADM formed from two 4x12 WSS. Transmission signals were generated by two Voyager white boxes each containing 4 transceivers. The Voyager receivers were used to receive and recover the signals. The bit error ratio (BER) performance of the recovered signals was obtained from the receiver diagnostics. The Voyager receivers were only able to recover Voyager signals. There was also spectral monitors to monitor both the receive and transmitted spectra.

UCL connected a broadcast and select ROADM to their fibre tails formed of a 50/50 coupler and a 1x4 WSS. The transmitted signals were generated by a 92 GS s$^{-1}$ DAC modulating two groups of external cavity lasers (ECLs). This allowed neighbouring channels to be decorrelated. Signals were received by a 30 GHz ICR connected to a 160 GS s$^{-1}$ four channel real time oscilloscope. The data was recovered and performance monitored using offline DSP implemented in MATLAB. The SNR of the recovered constellations was estimated by comparing the measured and expected constellation given knowledge of the transmitted bit sequence. They also had spectral monitors to monitor both the received and transmitted spectra.

**Figure 6:**
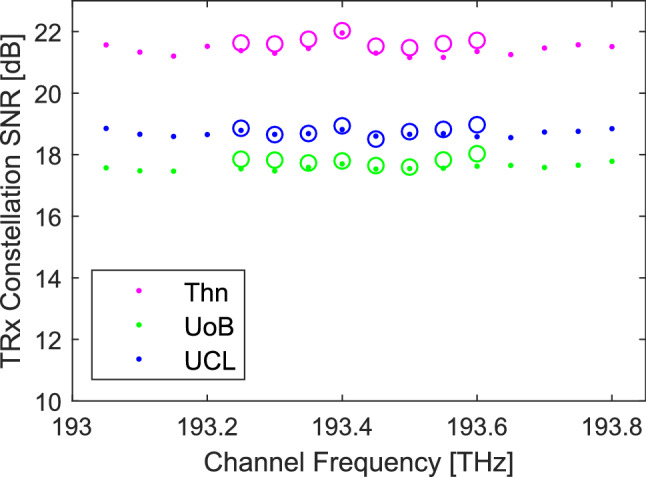
Channel performance. UoC measured optical channel performance for each channel, for the three loop backs and for 8 or 16 channels, $\approx$32 GBd PM-QPSK. Dots refer to 16 channel measurements, circles to 8 channel measurements^21^.

In measuring the NSR the performance for the loop-back paths has been taken as the average of the performance of the 8 channels. We have assumed that over the limited bandwidth used in these experiments the performance is independent of wavelength. For wider band operation it may be necessary to have different abstracted NSR for each wavelength or band of wavelengths where the launch power has been adjusted to give similar performance across the band. Figure 6 shows the measured network performance by UoC across all channels for the loop-back measurements to Thn, UoB and UCL. This is the performance of the optical channel with the back to back performance of the transceiver and its local node removed for 8 and 16 consecutive channels of $\approx$32 GBd PM-QPSK on a 50 GHz grid. There is a small fluctuation in performance across the channels with an average standard deviation of 0.15 dB, a 3% variation of NSR. It also shows on average the NSR increases by 4.5% for the 16 channel performance over the 8 channel performance slightly less than the GN predicted 5.5% but well within the experimental uncertainty.

**Table 6:**
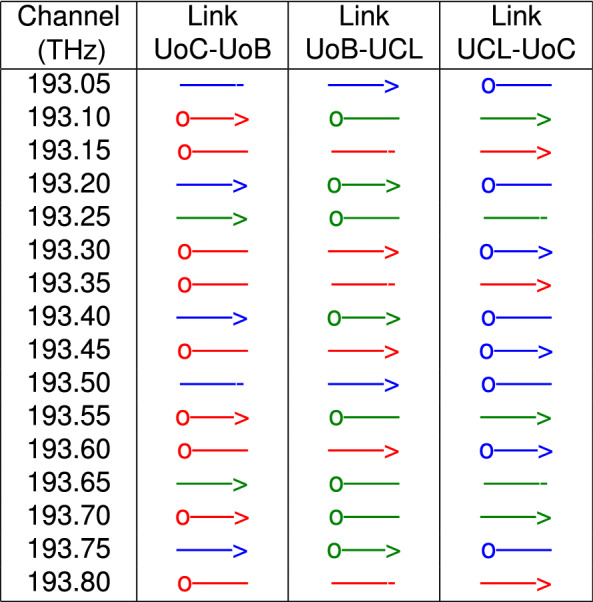
Routeing and channel assignment for virtual ring performance verification.

Signals propagate from “o—” left to right and loop round to “—>”. Colours designate signal origin.

**Figure 7:**
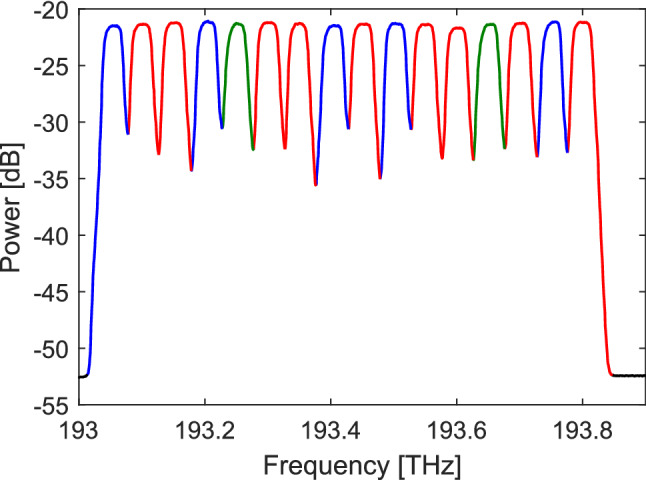
Spectrum. The outbound power spectrum from UoC, for the 16 channel, PM-16QAM, virtual ring verification measurements. Colours designate signal origin.

For the virtual ring network, Table 6 shows the routeing and wavelength allocation that was used. The launch spectrum from each partner was adjusted using the WSS channel attenuation to give a flat spectrum. Each partner in turn made adjustments with two rounds of adjustments being required to achieve a spectral flatness near ±0.5 dB. Figure 7 shows the launch spectrum from UoC. Since the OSA used were set to a high resolution much less than the symbol rate and given the slight variation in symbol rate between transmitters this adjustment results in a flat power spectral density being transmitted. This will lead to a similar SNR performance for small variations in the symbol rate.

### Receiver calibration

**Figure 8:**
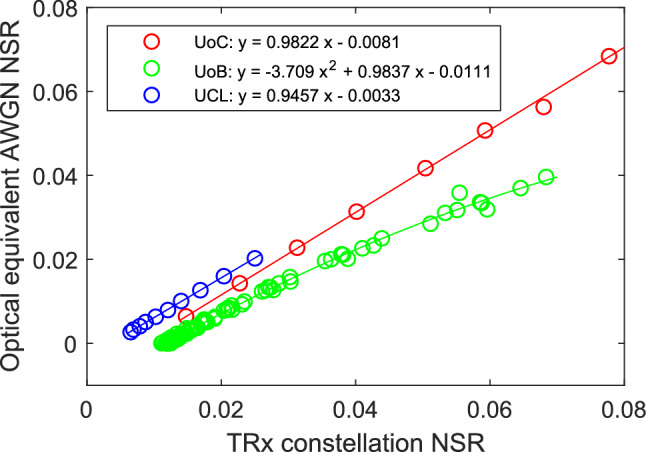
Transceiver calibration. Equivalent AWGN optical NSR vs TRx constellation measured NSR performance for the three partners^21^.

To give a consistent measure of the optical NSR within the network a noise loading calibration of each receiver was carried out. The NSR measured on an optical spectrum analyser for an equivalent noise bandwidth equal to the symbol rate was compared to the receiver measured performance. For UoC and UCL the receiver constellation based SNR was converted directly to NSR while for UoB the receiver measured BER was first converted to SNR based on the ideal error probability for 16QAM assuming only nearest neighbour constellation errors and ideal Gray coding. Figure 8 shows the noise loading calibration results for the three partners along with a least squares fit to allow direct translation from measured to calibrated NSR.

### WSS crosstalk

**Figure 9:**
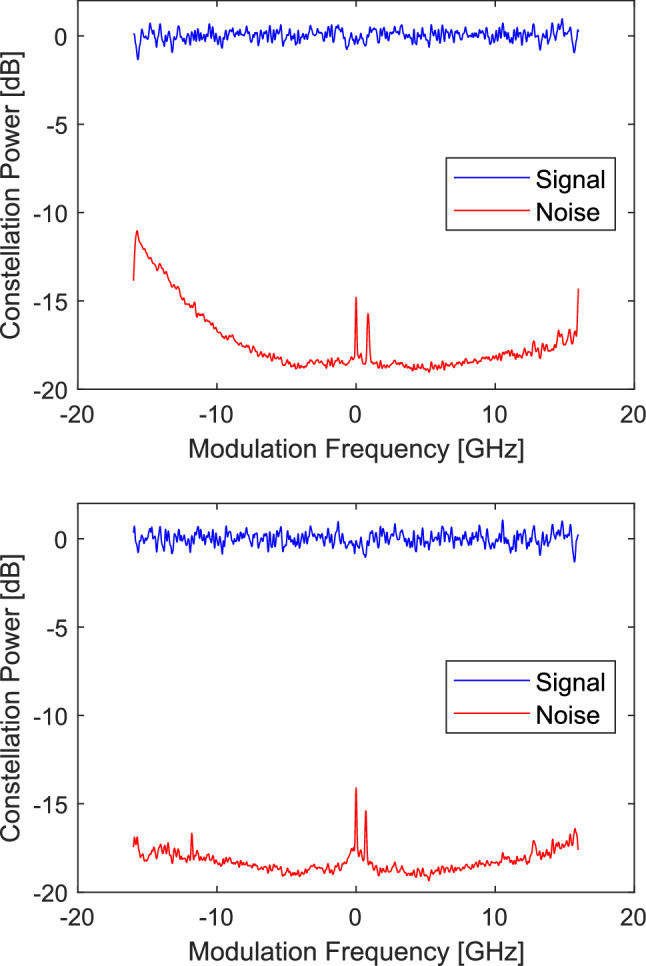
WSS interference. The spectrum of the signal and noise of the recovered constellation for the signal at 193.30 THz top and 193.45 THz bottom.

An investigation into the poor performing signals from UCL in Fig. 4 revealed an excess of noise in the low frequency side of the signal. Figure 9 shows the spectrum of the noise within the signal bandwidth, this has been obtained by taking the power spectrum of the error between the equalised symbols and the expected symbols. It can be seen that for the channel at 193.30 THz there is significant additional noise in the lower frequency spectrum equating to approximately 1.2 dB of extra noise, this is not observed for the channel at 193.45 THz, the centre channel of a routed group of three. This is caused by poor isolation of the WSS used in the ROADM node at UCL. The WSS gives poorer isolation at the switched channel edge, at UCL this combined with a small frequency offset in the switched channel centre leads to significant signal from the blocked channel being admitted with the intended channel. The cross talk was observed at UoC in the channels transmitted from UCL at 193.20, 193.30, 193.40, 193.60 and 193.75 THz. These channels have a switch channel edge on the lower frequency side and a signal in the channel below. Where a block of channels are switched together there is no switch channel edge within the block and excess noise was only observed on the lowest frequency channel, containing the low frequency switched channel block edge.

### BER estimation

For a given SNR at the constellation the BER can be predicted^22^, eq (2)] as

1 $$\begin{aligned} {\mathrm{BER}} \simeq A \ \mathrm{erfc}\left( \sqrt{B \ \mathrm{SNR}}\right) , \end{aligned}$$

where the constants *A* an *B* depend on the modulation format and are given in Table 7 and the function $\mathrm{erfc}(X)$ is the complimentary error function given by

2 $$\begin{aligned} \mathrm{erfc}(X) = \frac{2}{\sqrt{\pi }} \int _X^{\infty } \exp \left( -x^2\right) dx . \end{aligned}$$

The formula is inverted to convert the measure BER from the Voyagers at UoB into equivalent constellation SNR. For PM-QPSK modulation this form is exact for an AWGN channel, while for PM-16QAM modulation it is an approximation assuming only nearest neighbours errors and ideal Gray coding.

Similarly this Eq. (1) can be inverted to calculated the SNR above which an acceptable BER is obtained. This can then be used to define a threshold that the sum of NSR, along a path including the transmitter and receiver, must not exceed.

**Table 7:** Constant for use in Eq. (1) to estimate the BER for a AWGN channel.

Modulation format	A	B
PM-QPSK	$\mathsf{{1}\big /{2}}$	$\mathsf{{1}\big /{2}}$
PM-16QAM	$\mathsf{{3}\big /{8}}$	$\mathsf{{1}\big /{10}}$

## Supplementary information

Supplementary information.
