# Supplementary material for: Distributed abstraction and verification of an installed optical fibre network
Source: Sci Rep. 2021 May 24;11:10750. doi: 10.1038/s41598-021-89976-w (PMC8144218; doi:10.1038/s41598-021-89976-w)
Supplement: Supplementary file 1 — Supplementary information. [file 41598_2021_89976_MOESM1_ESM.pdf]

Supplementary Information to: Distributed Abstraction and Verification of an Installed Optical Fibre Network

D. J. Ives[1,*], S. Yan[2], L. Galdino[3], R. Wang[2], D. J. Elson[3,4], Y. Wakayama[3,4], F. J. Vaquero-Caballero[1], G. Saavedra[3,5], D. Lavery[3], R. Nejabati[2], P. Bayvel[3], D. Simeonidou[2], and S. J. Savory[1]

[1]University of Cambridge, Cambridge, UK
[2]University of Bristol, Bristol, UK
[3]University College London, London, UK
[4]KDDI Research, Inc., Fujimino, Japan
[5]Universidad de Concepción, Concepción, Chile
[*]Corresponding author, di231@cam.ac.uk

ABSTRACT

This supplement provides additional experimental detail of the design of the signal launch powers to give reasonable, near optimum SNR performance.

Network Configuration and Physical Layer Design

The National Dark Fibre Facility (NDFF) consists of a number of leased dark fibre spans connected by Polatis fibre space switches. The Polatis space switches allow monitoring of the output power and adjustment of the output power via an integrated VOA. Also connected to the Polatis switches were a number of EDFA and dispersion compensating modules (DCM). The DCM were not used, the network was designed as a coherent transmission dispersion uncompensated system.

Three types of EDFA were installed pre-amplifiers (PA), power amplifiers (BO) and inline amplifiers (IL) all types had input and output power monitors and had gain flattening designed to operate at a gain of 20 dB. The EDFA were operated in a constant gain mode where the pump current was controlled, based on the input and output power monitors and expected ASE noise, to ensure the design signal gain of 20 dB. All the EDFA had a similar noise figure, reviewing UoC data for the abstractions in the Summer of 2018[1] a noise figure of 5.75 dB explains the observed OSA traces. The PA and IL had a saturated output power of 15 dBm, while the BO could output 20 dBm.

Previous experience of operating NDFF[1,2] had confirmed that the installed fibre had an attenuation of $\approx$0.22 dB$\cdot$km^{-1}, a chromatic dispersion of $\approx$16.4 ps$\cdot$nm$^{-1}\cdot$km^{-1} and an effective nonlinear coefficient $\approx$1.16 W$^{-1}\cdot$km^{-1} where the term "effective" is used since the launch power was measured with the Polatis switch output monitor and will be slightly higher than the actual power propagated into the fiber.

The monitored total span loss, including connector and switch losses, A dB, and the EDFA noise figure, NF dB allow the calculation of the ASE noise power, σ^2_{ASE}, generated in a span with respect to the launch power, P, into the span as

$$\sigma^2_{ASE} = 10^{\frac{NF}{10}} h\nu 10^{\frac{A}{10}} R \tag{1}$$

where R is the symbol rate and is equal to the noise bandwidth after ideal matched filtering, $h = 6.626 \times 10^{-34}$ J$\cdot$s is Plank's constant and ν is the optical carrier frequency. Following the GN model[3,4] the nonlinear interference power is given by

$$\sigma^2_{NLI} = \eta P^3 \tag{2}$$

where η is a worst case nonlinear interference coefficient describing the NLI on the central channel under fully loaded conditions. To allow for coherent addition of the NLI over multiple spans η was calculated as $^1/_{16}$ of the value calculated for 16, the maximum possible, similar spans. The coefficient η was obtained by numerical integration of the GN model equation

$$\eta = \frac{16}{27}\gamma^2 \int_{-\infty}^{\infty}\int_{-\infty}^{\infty}\int_{-\infty}^{\infty} g(f_1)\, g(f_2)\, g(f_1+f_2-f)\, H(f)\, \rho(f_1,f_2,f)\, \chi(f_1,f_2,f)\, \mathrm{d}f_1\, \mathrm{d}f_2\, \mathrm{d}f, \tag{3}$$

where, in practice, the range of f is restricted to a single channel by $H(f)$, the range of f_1 and f_2 is restricted to the maximum extent of the transmitted comb, through $g(f_{1,2})$, and

$$\rho(f_1, f_2, f) = \frac{1 + \mathrm{e}^{-2L_s\alpha} - 2\mathrm{e}^{-L_s\alpha}\cos\left[4\pi^2\beta_2(f_1-f)(f_2-f)L_s\right]}{\alpha^2 + \left[4\pi^2\beta_2(f_1-f)(f_2-f)L_s\right]^2}, \tag{4}$$

$$\chi(f_1, f_2, f) = \frac{\sin^2\left[2\pi^2\beta_2(f_1-f)(f_2-f)L_sN_s\right]}{\sin^2\left[2\pi^2\beta_2(f_1-f)(f_2-f)L_s\right]}, \tag{5}$$

N_s is the number of spans, L_s is the span length, α is the fibre attenuation coefficient, β_2 is the fibre chromatic dispersion coefficient and γ is the fibre nonlinear coefficient. For practical signal spectra the integration of f is over the central channel matched filter while f_1 and f_2 is over the comb of signal channels. The integration was carried out numerically using the importance sampled Monte-Carlo technique.

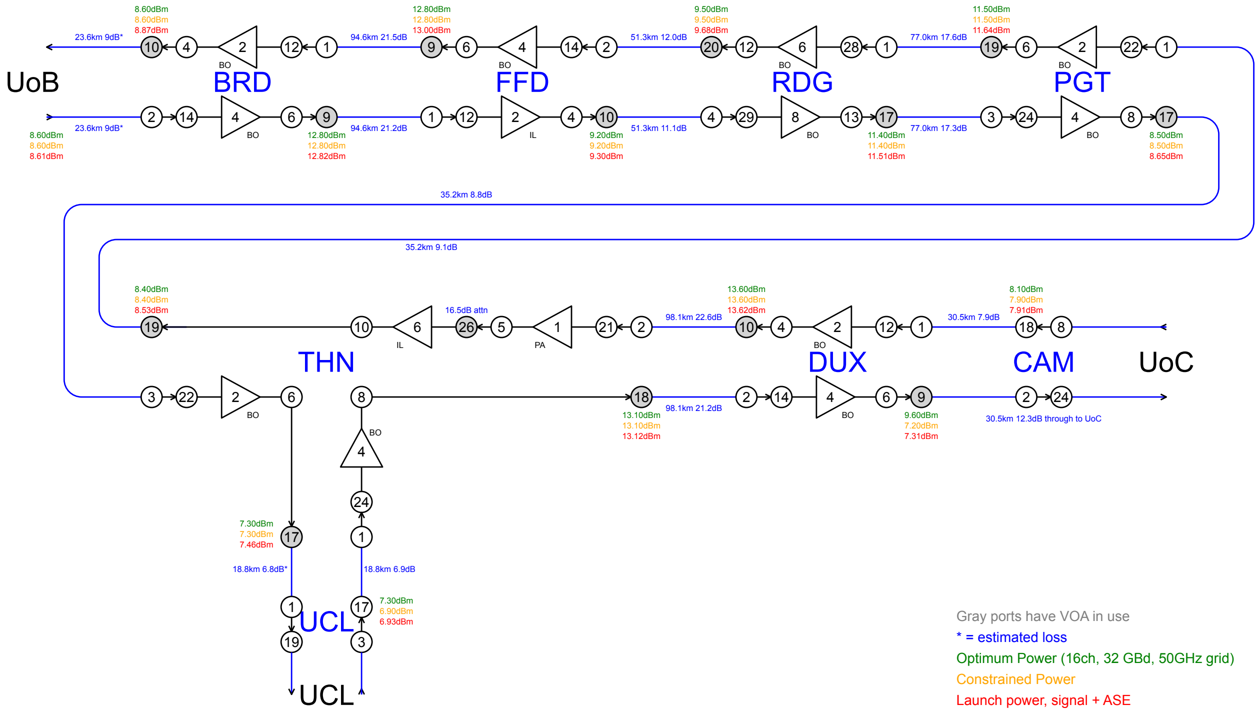

Figure 1. NDFF detailed setup. Connections between components of NDFF used to create the physical topology. The launch power into each span is marked.

The channels were assumed to have a symbol rate, R, of 32 GBd, with an ideal rectangular spectrum and a WDM grid spacing, Δf of 50 GHz. For the full load of $N_{ch} = 16$ channels the worst NLI occurs on a central channel. The signal power spectral density $G(f) = p/g(f)$ where $g(f)$ is the spectral shape given as

$$g(f) = \frac{1}{R}\sum_{i=\left\lceil -\frac{N_{ch}-1}{2}\right\rceil}^{\left\lceil \frac{N_{ch}-1}{2}\right\rceil} \Pi\left(\frac{f+i\Delta f}{R}\right) \tag{6}$$

where $\Pi(x)$ is the rectangle function defined as $\Pi(x) = 0$ for $|x| > 1/2$, $\Pi(x) = 1/2$ for $|x| = 1/2$ and $\Pi(x) = 1$ for $|x| < 1/2$.
while the match filter $H(f)$ was

$$H(f) = \Pi\left(\frac{f}{R}\right). \tag{7}$$

Finally we have the SNR of a span is given as

$$SNR = \frac{P}{\sigma^2_{ASE} + \sigma^2_{NLI}}. \quad (8)$$

This leads to an optimum launch power P_{opt} given by

$$P_{opt} = \sqrt[3]{\frac{\sigma^2_{ASE}}{2\,\eta}}. \quad (9)$$

In the case where the noise from each span is independent then the global optimum link SNR occurs when each span is operated at its local optimum. This is the LOGO principle[5].

In order to ensure that the EDFA all operate at their design gain and give flat gain performance the EDFA must not reach saturation. As such the input power to PA and IL models must not exceed -5 dBm, while the input to BO models must not exceed 0 dBm. Where with short spans with low attenuation this input requirement would be exceeded the launch power into those spans was restricted. Finally the accumulation of ASE noise was added to the signal power. The table 1 shows the span loss, η, optimum launch power per channel, total optimum power for 16 channels, total constrained power and total constrained power + ASE noise.

ID	Length [km]	Loss [dB]	η [mW^{-2}]	P_{opt} [dBm]	Total Optimum Signal Power [dBm]	Constrained Signal Power [dBm]	Constrained Signal and ASE Power [dBm]
Cam-Dux	30.5	7.9	0.00071	-3.9	8.1	7.9	7.91
Dux-Thn	98.1	22.6	0.00062	1.6	13.6	13.6	13.62
Thn-Pgt	35.2	8.8	0.00072	-3.6	8.4	8.4	8.53
Pgt-Rdg	77.0	17.6	0.00065	-0.6	11.5	11.5	11.64
Rdg-Ffd	51.3	12.0	0.00070	-2.5	9.5	9.5	9.68
Ffd-Brd	94.6	21.5	0.00062	0.8	12.8	12.8	13.00
Brd-UoB	23.6	9.0	0.00068	-3.5	8.6	8.6	8.87
UoB-Brd	23.6	9.0	0.00068	-3.5	8.6	8.6	8.61
Brd-Ffd	94.6	21.2	0.00062	0.7	12.8	12.8	12.82
Ffd-Rdg	51.3	11.1	0.00070	-2.8	9.2	9.2	9.30
Rdg-Pgt	77.0	17.3	0.00065	-0.6	11.4	11.4	11.51
Pgt-Thn	35.2	9.1	0.00072	-3.5	8.5	8.5	8.65
Thn-UCL	18.8	6.8	0.00063	-4.1	7.9	7.3	7.46
UCL-Thn	18.8	6.9	0.00063	-4.1	7.9	6.9	6.93
Thn-Dux	98.1	22.3	0.00062	1.1	13.1	13.1	13.12
Dux-Cam	30.5	12.3	0.00071	-2.4	9.6	7.2	7.31

Table 1. Design launch powers into each span for 16 channels of ≈32 GBd on a 50 GHz grid.

Figure 1 shows the layout of elements within NDFF. Circles represent Polatis switch ports, grey colored launch ports make use of the integral VOA and are annotated with the design launch power for 16 channels.

Acknowledgements

The authors acknowledge funding support from the UK EPSRC (through the project INSIGHT EP/L026155/2 and the programme TRANSNET EP/R035342/1)), the UK Royal Academy of Engineering under the Research Fellowships scheme and the EU H2020 project(Metro-Haul,761727), equipment from Ciena and Facebook and use of the UK EPSRC National Dark Fibre Facility, EP/S028854/1.

References

1. Ives, D. J. et al. A Comparison of Impairment Abstractions by Multiple Users of an Installed Fiber Infrastructure. In Optical Fiber Communication Conference (OFC) 2019, 1, M4J.4, DOI: 10.1364/OFC.2019.M4J.4 (San Diego, CA. (USA), 2019).

2. Ives, D. J., Vaquero Caballero, F. J. & Savory, S. J. Remote Abstraction of an Installed Dark Fiber Network using Noise to Signal Ratio. In Optical Fiber Communication Conference, DOI: 10.1364/OFC.2018.Tu3E.2 (2018).

3. Poggiolini, P. The GN Model of Non-Linear Propagation in Uncompensated Coherent Optical Systems. J. Light. Technol. **30**, 3857–3879, DOI: 10.1109/JLT.2012.2217729 (2012).

4. Ives, D. J., Bayvel, P. & Savory, S. J. Adapting Transmitter Power and Modulation Format to Improve Optical Network Performance Utilizing the Gaussian Noise Model of Nonlinear Impairments. J. Light. Technol. **32**, 3485–3494, DOI: 10.1109/JLT.2014.2346582 (2014).

5. Poggiolini, P. et al. The LOGON Strategy for Low-Complexity Control Plane Implementation in New-Generation Flexible Networks. In Optical Fiber Communication Conference, 1, OW1H.3, DOI: 10.1364/OFC.2013.OW1H.3 (Anaheim, CA. (USA), 2013).
